# Canagliflozin reverses Th1/Th2 imbalance and promotes podocyte autophagy in rats with membranous nephropathy

**DOI:** 10.3389/fimmu.2022.993869

**Published:** 2022-12-01

**Authors:** Xin Lv, Jian Wang, Li Zhang, Xian Shao, Yao Lin, Hongyan Liu, Guangyang Ma, Jing Li, Saijun Zhou, Pei Yu

**Affiliations:** ^1^ NHC Key Laboratory of Hormones and Development, Chu Hsien-I Memorial Hospital and Tianjin Institute of Endocrinology, Tianjin Medical University, Tianjin, China; ^2^ Tianjin Key Laboratory of Metabolic Diseases, Tianjin Medical University, Tianjin, China; ^3^ Department of Nephrology, Shanxi Bethune Hospital, Shanxi Academy of Medical Sciences, Tongji Shanxi Hospital, Third Hospital of Shanxi Medical University, Taiyuan, China; ^4^ Tongji Hospital, Tongji Medical College, Huazhong University of Science and Technology, Wuhan, China; ^5^ Department of Nephrology, First Affiliated Hospital of Gannan Medical University, Ganzhou, China

**Keywords:** membranous nephropathy (MN), canagliflozin, T-cells, B-cells, podocyte autophagy

## Abstract

Idiopathic membranous nephropathy is the main cause of chronic kidney disease (CKD). Studies have shown sodium–glucose co-transporter 2 (SGLT2) inhibitors significantly delay renal outcomes in patients with CKD, but the exact mechanism remains unknown. In this study, we investigated the mechanism by which the SGLT2 inhibitor canagliflozin attenuates podocyte injury by reversing the imbalance in Helper T cell 1 (Th1)/Helper T cell 2 (Th2) in peripheral blood of rats with membranous nephropathy (MN). MN rats were gavaged with canagliflozin (10 mg/kg/d) and losartan (10 mg/kg/d), respectively, for eight weeks. Compared with the MN group, the urinary ratio of total protein and the creatinine levels of the canagliflozin group decreased significantly. Canagliflozin improved the glomerulus pathological damage, increased the expression levels of podocyte marker proteins. The protective effect of canagliflozin on kidneys was more obvious than that of losartan. Treatment with canagliflozin increased the proportion of Th1 cells by 2.3 times, decreased the proportion of Th2 cells by 68.5%, and significantly restrained the synthesis of immunoglobulin G1 in B-cells and glomerulus subepithelial immune complex deposition. Co-culture of B-cells derived from MN rats with podocytes triggered the activation of phosphorylation of mTOR and ULK1 of podocytes, inhibited podocyte autophagy and resulted in podocyte injury. B-cells derived from canagliflozin treatment rats reversed these effects above. In conclusion, canagliflozin exerts a protective effect on kidneys by reversing the imbalance in Th1/Th2 cells in MN rats and restoring the autophagy of podocytes inhibited by the abnormal immunoglobulin G secretion from B-cells.

## Introduction

Idiopathic membranous nephropathy (IMN), an organ-specific autoimmune disease, is one of the most common causes of adult nephrotic syndrome and one of the main causes of end-stage renal disease (1–3). In recent years, the incidence of IMN has risen sharply. A retrospective study from China, which analyzed 34,630 hospitalized patients who underwent renal biopsy between January 1, 2009, and December 31, 2018, confirmed that MN (24.96%) has surpassed IgA nephropathy (24.09%) as the most common pathological type of primary glomerulonephritis in Chinese adults (4). Initial therapy for patients with membranous MN is supportive (5); immunosuppressive therapy is recommended for patients with persistent nephrotic syndrome (5, 6). However, immunosuppressive therapy can significantly increase the risk of clinical toxic effects, including hyperglycemia, myelosuppression, infections, infertility, and cancer (7, 8). RAS inhibitors can only alleviate proteinuria partly thus ineffectively inhibiting the progression of IMN (9). Therefore, there is an urgent need to seek a treatment for IMN that takes into account the concepts of effectiveness, safety, economy, and simplicity.

IMN is a glomerulonephritis characterized by podocyte injury mediated by antibodies against podocyte antigens deposited under the glomerular visceral epithelial cells to activate the complement. The pathological manifestations of the kidneys are diffuse thickening of the glomerular basement membrane, spike structures, massive deposition of electron-dense material under epithelial cells, and granular deposition of IgG and C3 along the capillary wall. The deposition of immune complexes plays a key role in the pathogenesis of IMN. Helper T cell 17 (Th17)/regulatory T (Treg) was reported to take part in the development of IMN and the mechanism tends to indirectly affect B cells and humoral immunity by regulating inflammation (10–12), Th17 was also associated with thrombosis and relapses in patients with membranous nephropathy (13). Compared with Th17, Th2 is more directly associated with humoral immune disorders in membranous nephropathy and Th1/Th2 polarization imbalance plays an important role in the pathogenesis of IMN (14–16). Since IgG extracted from the serum of LN patients not only induces podocyte apoptosis but also affects podocyte autophagy (17, 18), We speculate that a Th1/Th2 polarization imbalance may lead to abnormal IgG secretion by B-cells, which may result in podocyte autophagy dysfunction and podocyte injury.

Sodium-glucose co-transporter 2 (SGLT2) inhibitors, a newly listed type of hypoglycemic drug, not only effectively lower blood glucose levels and improve cardiovascular risk factors of diabetic patients (i.e., reducing weight, blood pressure, and blood lipid and uric acid levels) but also effectively delay the disease progression of diabetic patients with CKD (19, 20). The CREDENCE study focused on the renal outcomes of diabetic patients as the primary endpoint and confirmed that canagliflozin significantly reduced the risk of the renal composite hard endpoint (ESKD, serum creatinine doubling, and renal or cardiovascular death) in diabetic patients by 30% and consistently reduced urinary protein levels by up to 31% (21). In addition, the results of a subgroup analysis according to glycated hemoglobin HbA1c level in this study revealed that the reno-protective effect of canagliflozin in diabetic patients with CKD was independent of its hypoglycemic effect (21). The results of this analysis strongly suggest a potential therapeutic effect of canagliflozin in non-diabetic proteinuria as well. Our previous study evaluated the early reno-protective effect of canagliflozin in newly diagnosed type 2 diabetic patients using multimodal magnetic resonance imaging. It was found that canagliflozin can improve renal oxygenation, which may be independent of its hypoglycemic effect and tubuloglomerular feedback (22). In addition, the Dapagliflozin And Prevention of Adverse outcomes in Chronic Kidney Disease (DAPA-CKD) study further confirmed, in a non-diabetic CKD population (with chronic glomerulonephritis, e.g., IgA nephropathy), that dapagliflozin significantly reduced the risk of renal-specific endpoints (i.e., sustained decline in estimated glomerular filtration rate ≥ 50%, development of end-stage renal disease or renal death) by 44% (23, 24). Therefore, SGLT2 inhibitors may reduce proteinuria and delay the progression of nephropathy in CKD patients by mechanisms distinct from those other than tubuloglomerular feedback. SGLT2 inhibitors were reported to have immunomodulatory effects which improved the Th17/Treg cell imbalance in diabetic mice (25). Abnormal secretion of IgG from homologous B cells mediated by Th2 is an important mechanism for membranous nephropathy. Whether SGLT2 inhibitors could reverse the imbalance of Th1/Th2 and suppress the humoral immune induced by B cells is unknown.

In the present study, we observed the improvement of urinary protein and renal histopathology in MN rats driven by canagliflozin and further investigated the possible mechanism of its action in improving the Th1/Th2 imbalance and inhibiting the podocyte injury mediated by abnormal IgG secretion from B-cell in MN rats, providing new ideas for the treatment of IMN.

## Materials and methods

### Animal models and experimental groups

Male Sprague–Dawley rats (180–200 g) were purchased from Spelford Biotechnology Co. (Beijing, China). The animals were housed in a specific pathogen-free room at 20°C–25°C and fed ad libitum with water and a standard diet in a continuous 12:12-h light–dark cycle. After 1 week of adaptive housing, the rats were randomly divided into a modeling group and a control group. The MN rat model was induced using cationic bovine serum albumin (C-BSA) for a total of 5 weeks. In the first week, the modeled rats were injected subcutaneously with 1 mg of C-BSA and an equivalent amount of Freund’s incomplete adjuvant daily. After that, they were injected with 16 mg/kg of C-BSA into the tail vein every other day for 4 weeks (15 times), and the control group was given saline alone at the same time. Body weight, urine glucose, 24-hour urine protein, and urine creatinine were measured weekly during the modeling period. Blood and urine biochemical tests were performed at baseline and week 5, and kidney tissues from three rats in the modeling group were randomly selected for pathological examination. Subsequently, 24-hour urine protein quantification > 100 mg and electron microscopy findings suggested a thickened basement membrane and subepithelial electron dense deposits, indicating successful modeling. After that, the rats were divided into 4 groups (n = 6 each group), i.e., a normal group, model group, canagliflozin group, and losartan group. All experimental rats received gavage administration for 8 weeks. The rats in the model group were given distilled water as a control, and the rats in the canagliflozin and losartan groups were treated with 10 mg/kg/d of canagliflozin or 10 mg/kg/d of losartan, respectively, as previously reported (26, 27). During the treatment period, all rats were evaluated weekly for body weight and every 4 weeks regarding 24-h total urine protein, urine creatinine, urine glucose, blood biochemistry, and other indices. At the end of the eighth week of treatment, the rats were sacrificed, and renal tissues were collected for further analysis.

### Serum and urine measurements

We collected 24-h urine specimens using rat metabolic cages, and 24-h urine protein quantification and urine creatinine and urine glucose levels were detected using an immunoturbidimetric method. Blood was collected from the rat’s inner canthus, and an AU5800 automatic biochemical analyzer (Beckman Coulter, USA) was used to detect biochemical indices, such as blood glucose, blood creatinine, blood uric acid, plasma albumin, and blood lipid levels. Blood and urine indexes were tested at the Department of Laboratory of Chu Hsien-I Memorial Hospital of Tianjin Medical University.

### Periodic acid-sliver methenamine staining

Kidney tissues were fixed in 4% paraformaldehyde for 48 h, then paraffin-embedded and sectioned. Subsequently, paraffin sections were dewaxed to water, processed for PASM staining, dehydrated, and sealed, and the histopathological changes in kidney tissues were observed under a microscope.

### Transmission electron microscopy

For electron microscopy examinations, small pieces (1 mm3) of kidney tissue were fixed in 2.5% glutaraldehyde in 0.1 M of sodium cacodylate buffer (pH, 7.4), with 1% (wt/vol) osmium tetroxide, and embedded in epoxy resin. Ultra-thin sections (0.1 μm thick) were double-stained with uranyl acetate and lead citrate and examined with an electron microscope. Transmission electron micrographs were obtained using a JEM-1400 Flash electron microscope (JEOL Ltd., Tokyo, Japan) operated at 60 kV with an absolute magnification of ×8000.

### Immunofluorescence analysis

Frozen sections of 2-μm-thick kidney tissue were fixed in acetone, blocked with 1% bovine serum albumin in phosphate-buffered saline (PBS), and incubated with primary anti-rat IgG (31220; Invitrogen, USA), C3 (HYB 118-02-02; Invitrogen, USA), and podocin (20384-1-AP; Proteintech, China) at 4°C, followed by FITC-conjugated secondary antibody at 37°C for 1 h before being examined under a IX71 fluorescence microscope (Olympus Corp., Tokyo, Japan). In the same way, frozen sections of kidney tissue were incubated with the FITC-conjugated primary antibodies IgG1 (clone R1-12D10, anti-rat; Invitrogen, USA) and IgG2a (clone 2A 8F4, anti-rat; Abcam, UK), which was followed by direct immunofluorescence microscopy.

### Confocal microscopic observation of suspended T-cells

T-cells were sorted from rat peripheral blood mononuclear cells (PBMCs) by sorting flow cytometry (Aria II) using PerCP/Cyanine5.5 combined with anti-rat CD3 (201417; Biolegend, USA), fixed in 4% paraformaldehyde for 30 min, then subsequently incubated at 37°C with the primary antibody to anti-rat SGLT2. After rinsing, T-cells were incubated with goat anti-rabbit IgG(H+L)-HRP with FITC for 1 h at 37°C. After rinsing, T-cells were resuspended in PBS, adhered to poly-L-lysine–coated slides, and treated with a DAPI solution containing a blocker (no. S2110; Solabao, Beijing, China) to stain the nuclei. We added coverslips, then observed the cells with an LSM800 laser scanning confocal microscope (Zeiss, Jena, Germany).

### Peripheral blood PBMC preparation and flow cytometry analyses

Blood samples were taken from the retro-orbital plexus of rats, and 1.5 mL was collected in heparin tubes and mixed upside down. PBMCs were isolated by Ficoll–Hypaque gradient centrifugation. Flow cytometry was used to determine the phenotype of each group of rat T-cells, B-cells, and Th1 and Th2 cells using various combinations of the following fluorochrome-conjugated monoclonal antibodies: perCP/Cyanine5.5-conjugated anti-rat CD3 (201417; Biolegend, USA), FITC-conjugated anti-rat CD4 (130-107-667; Miltenyi, Germany), eFluor 450–binding anti-rat CD8 (48-0084-80; Thermo Fisher Scientific, USA), PE-CYN7–binding anti-rat CD45R (25-0460-80; Thermo Fisher Scientific, USA), Alexa Fluor^®^ 647-binding anti-rat interferon (IFN)-γ (507809; Biolegend, USA), and PE-conjugated anti-rat interleukin (IL)-4 (511905; Biolegend, USA) for multicolor immunofluorescence (IF).

To analyze intracellular cytokine production, PBMCs were stimulated with 1 μL/mL of eBioscience cell-stimulation cocktail (composition: phorbol 12-myristate 13-acetate, lonomycin, brefeldin A, monensin in Ethano. 00-4975-93; Thermo Fisher Scientific, USA) stimulated PBMCs for 4 h. For intracellular staining, cell surface antigens were first stained, followed by fixation with FIX & PERM KIT (GAS003; Invitrogen, USA), membrane breaking, followed by staining for intracellular cytokines IFN-γ and IL-4 with fluorochrome-conjugated monoclonal antibodies. The stained cells were washed with PBS, and FACS data were collected by a Fortessa high-end analytical flow cytometer (BD LSRFortessa, USA) and analyzed using the FlowJo software.

### Magnetic bead isolation of B-cells and *in vitro* culture expansion

Peripheral blood mononuclear cells (PBMCs) were isolated from each group of rat peripheral blood using the Ficoll–Hypaque gradient centrifugation method. B-cells were further purified using the negatively selected B-cell enrichment kit EasySep™ rat B cell isolation kit (19644; StemCell Technologies, Canada) according to the manufacturer’s procedures. All samples were ≥97% pure.

Freshly isolated B-cells were cultured using 24-well plates in RPMI 1640 medium supplemented with 10% inactivated FCS, 2 mM L-glutamine, 200 U/mL of penicillin, 100 mg/mL of streptomycin, and 10 Mm of HEPEs. B-cells were cultured on the first day of culture using CD40 precoated 24-well plates (Costar 24-well Clear TC-treated plates), 10 μg/mL of LPS (L4391; Sigma-Aldrich, St. Louis, MO, USA) and 50 ng/mL of IL-4 (400-04; PEPROTECH, China) were added on the first day of B-cell culture, and B-cell supernatants were collected after 12 days of *in vitro* culture.

### Detection of anti-IgG1 and anti-IgG2 antibodies by ELISA

The rat B-cell culture supernatant of each group was diluted 1:5 in sample buffer and incubated for 30 min. After 3 washes with wash buffer, the bound antibodies were detected by incubating with anti-rat IgG1 or anti-rat IgG2a HRP conjugates for 30 min. The samples were then washed again as described previously with the addition of tetramethylbenzidine (TMB) substrate for 10 min. All operations were performed at 25°C and the optical density (OD) was read at 450 nm within 15 min using an automated spectrophotometer.

### B-cell and mouse podocytel Transwell co-culture

Transwell cell culture inserts (well size: 0.4 μm; Corning Costar) were placed in RPMI 1640 medium with both upper and lower chambers containing 10% inactivated FBS and 1% double antibodies. *In vitro* expanded B-cells cultured for 12 days were resuspended at a density of 5×10^5^/well in the lower chamber (6-well plate), which contained both 10 μg/mL of LPS and 50 ng/mL of IL-4. Mouse podocytes (MPC-5) (C2046; WHELAB BIOSCIENCE LIMITED, Shanghai, China) were transferred from 33°C to 37°C for 3 days and resuspended at a density of 5×10^5^/well in the upper chamber, and cells were grown adnexally to 60%–70% at 48 h. Cytokines and metabolites produced by B-cells in the lower chamber were delivered through a polyester membrane. The control group was MPC-5 cultured in normal RPMI 1640 medium (without insert) placed in 6-well plates. The experimental group consisted of co-cultured MPC-5 and rat B-cells from the normal group, rat B-cells from the MN group, rat B-cells from the canagliflozin group, or rat B-cells from the losartan group, respectively.

### Western blotting analysis

Proteins were isolated from kidney tissue and MPC-5, and protein levels were assayed. Briefly, protein extracts were boiled in RIPA buffer (Beyotime, Shanghai, China) and separated by SDS-PAGE electrophoresis, transferred to NC membranes, closed, and then incubated overnight at 4°C with the primary antibodies nephrin (NPHS1 A3048; ABclonal, China), podocin (NPHS2 20384-1-AP; Proteintech, China), and synaptopodin (A8484; ABclonal, China) to detect their protein levels in kidney tissue and MPC-5. Beclin1 (A17028; ABclonal, China), p62 (A11250; ABclonal, China), LC3B (A19665; ABclonal, USA), Phospho-mTOR-S2448 (AP0094, ABclonal, China), mTOR (AF6308, Affinity, China), Phospho-ULK1-S757 (AP0736, ABclonal, China) and ULK1 (AF4687, Affinity, China) antibodies were assessed to detect their protein levels in MPC-5. GAPDH (AC002; Abclonal, China) or Tubulin (A17913; Abclonal, China) were used as reference proteins. The blots were then incubated with HRP-conjugated secondary antibodies for 1 h at room temperature and visualized using the Bio-Rad Gel Doc EZ imaging system (Bio-Rad Laboratories, Hercules, CA, USA) and enhanced chemiluminescence reagents (Millipore, Boston, MA, USA). All western blot analyses were performed ≥3 times and protein bands were quantified using the ImageJ software (U.S. National Institutes of Health, Bethesda, MD, USA).

### Statistical analysis

Results are presented as means ± standard error of the mean (SEM), derived from at least 3 independent experiments. The PRISM version 8 (GraphPad Software, San Diego, CA, USA) and SPSS version 25 (IBM Corporation, Armonk, NY, USA) statistical software programs were utilized for all statistical analyses. The Student’s t test was employed to evaluate the significant difference between the two independent groups. Comparisons among groups were performed using one-way analysis of variance (ANOVA) followed by Tukey’s test. P < 0.05 was considered statistically significant.

## Results

### Weight and biochemical indexes

The weight and biochemical indexes of the experimental rats are summarized in **Table 1**. There were no significant differences in baseline weight, blood urea nitrogen (BUN), serum creatinine (Scr), serum uric acid (SUA), triglyceride (TG), total cholesterol (TCHO), blood glucose, or urine sugar values among all groups (all P > 0.05). At week 8 post modeling, the weight of MN rats was significantly less than that of the control group (P < 0.01), and the blood TG and CHO levels increased significantly after modeling (P < 0.01). The Scr concentration in the MN group was higher than that in the control group after 8 weeks of modeling (P < 0.01). Urine glucose excretion of the canagliflozin group was significantly higher than that of other groups after 8 weeks of treatment (P < 0.01), while there was no statistical difference in blood glucose level among the groups. There was no statistical difference in body weight, BUN, Scr, SUA, TG, or CHO between the canagliflozin group and losartan group.

**Table 1:** Weight and biochemical test results of rats in each group.

	NC (*n *= 6)			MN (*n *= 6)			Canag (*n *= 6)			Los (*n *= 6)		
	baseline	Week 0	Week 8	baseline	Week 0	Week 8	baseline	Week 0	Week 8	baseline	Week 0	Week 8
Body weight(g)	182 ± 2.03	224 ± 2.43	598.6 ± 3.39	180.4 ± 2.47	215.8 ± 3.22	506.6 ± 26.54^B^	185.6 ± 3.76	213.7 ± 2.59	499.2 ± 16.73^B^	78.3 ± 2.19	218 ± 5.20	520.7 ± 11.16^A^
BUN(mM)	5.68 ± 0.21	5.84 ± 0.23	6.48 ± 0.3	5.96 ± 0.37	5.92 ± 0.49	6.5 ± 0.42	5.49 ± 0.33	5.5 ± 0.27	6.48 ± 0.44	5.49 ± 0.31	5.46 ± 0.49	6.44 ± 0.35
Scr(uM)	21.97 ± 1.96	23.78 ± 2.07	26.18 ± 2.24	23.45 ± 2.05	23.94 ± 3.11	42.98 ± 1.48^B^	21.45 ± 1.85	23.57 ± 2.33	38.33 ± 2.49^B^	23.54 ± 2.45	23.38 ± 3.2	40.9 ± 2.13^B^
SUA(uM)	63.02 ± 2.45	67.18 ± 3.75	79.86 ± 1.53	65.2 ± 2.07	80.7 ± 1.99^A^	84.36 ± 5.59	63.79 ± 1.08	78.47 ± 3.5	83.72 ± 4.87	67.94 ± 1.97	80.86 ± 2.95	83.16 ± 8.46
TG(mM)	0.81 ± 0.12	1.63 ± 0.21	1.09 ± 0.15	0.9 ± 0.08	6.38 ± 2.2^B^	4.96 ± 0.02^B^	0.89 ± 0.17	6.36 ± 1.05^A^	1.46 ± 0.32^D^	0.77 ± 0.08	6.32 ± 1.25^A^	2.34 ± 0.71^C^
TCHO(mM)	2.32 ± 0.17	2.4 ± 0.21	2.31 ± 0.21	2.58 ± 0.29	10.12 ± 1.5^B^	7.08 ± 1.01^B^	2.46 ± 0.12	10.53 ± 0.79^B^	3.04 ± 0.36^D^	2.33 ± 0.26	10.43 ± 1.5^B^	3.58 ± 0.76^D^
Blood glucose(mM)	5.85 ± 0.23	5.68 ± 0.16	6.14 ± 0.11	5.7 ± 0.19	5.86 ± 0.24	6.04 ± 0.42	5.61 ± 0.28	5.84 ± 0.14	6 ± 0.24	5.79 ± 0.32	5.75 ± 0.2	5.91 ± 0.2
Urine sugar(mM)	2.44 ± 0.33	2.73 ± 0.19	2.45 ± 0.15	2.72 ± 0.22	3.01 ± 0.3	2.58 ± 0.35	2.86 ± 0.21	2.95 ± 0.13	77.22 ± 1.08^BD^	2.69 ± 0.23	2.87 ± 0.19	2.81 ± 0.25

BUN: blood urea nitrogen; Scr: serum creatinine; SUA: serum uric acid; TG: triglyceride; TCHO: total cholesterol. Data are shown as mean ± SEM values. One-way ANOVA followed by Tukey’s test for multiple comparisons was used. ^A^P < 0.05, ^B^P < 0.01 versus NC; ^C^P < 0.05, ^D^P < 0.01 versus MN.

### Canagliflozin decreases proteinuria and improves renal pathological damage in MN rats

**Figure 1:**
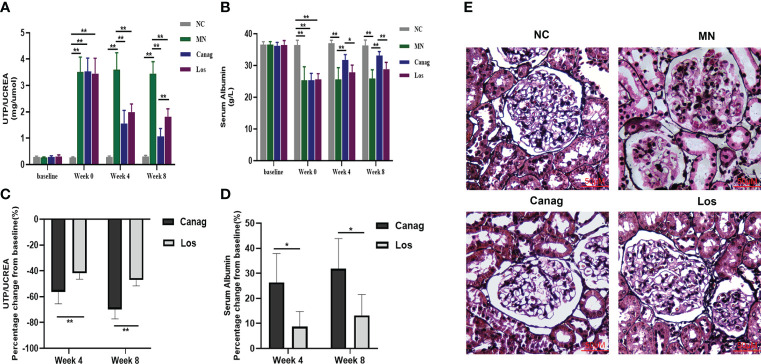
Canagliflozin improves the kidney disease of MN rats. **(A, B)** Urinary total protein/creatine and serum albumin at weeks 4 and 8 in membranous nephropathy rats treated with Canag and Los (n=6). Week 0 data refers to the data collected after modeling and before treatment. **(C, D)** Percentage change in urinary protein/creatine and serum albumin from baseline. **(E)** Representative PASM images of glomeruli. Scale bar, 50 μm. Data are shown as mean ± SEM values. The Student’s t test was employed to evaluate the significant difference between the two independent groups. One-way ANOVA followed by Tukey’s test for multiple comparisons was used. *P < 0.05, **P < 0.01.

In this study, the effect of canagliflozin on proteinuria in MN rats was first examined. Compared with NC rats, the urinary total protein/creatinine (mg/umol) was significantly higher in the rats after modeling (0.26 ± 0.01 vs. 3.51 ± 0.23, P < 0.01). Compared to MN rats, the urinary total protein/creatinine level decreased by 56.3% (P < 0.01) and 69.8% (P < 0.01), respectively, after 4 and 8 weeks of treatment by canagliflozin, and decreased by 42% (P < 0.01) and 47.1% (P < 0.01) in the losartan treatment group, as shown in **Figure 1A**. Serum albumin increased by 26.3% (P < 0.05) and 31.8% (P < 0.05) respectively in the canagliflozin treatment group, and there was a trend of increasing albumin levels in the losartan-treated group, but no statistical difference was reached (**Figure 1B**). No matter the decrease in urinary total protein/creatinine levels or the increase in the levels of serum albumin, canagliflozin was significantly better than losartan (**Figures 1C, D**). Compared to the MN group, the hyperplasia of glomerular mesangial cells and stroma, the thickening of basement membrane and spiky structure were improved in the 2 groups after eight weeks of treatment. The improvement degree of the canagliflozin group was better than that of the losartan group (**Figure 1E**).

### Canagliflozin reduces renal immune complex deposition and improves podocyte injury in rats with membranous nephropathy

**Figure 2:**
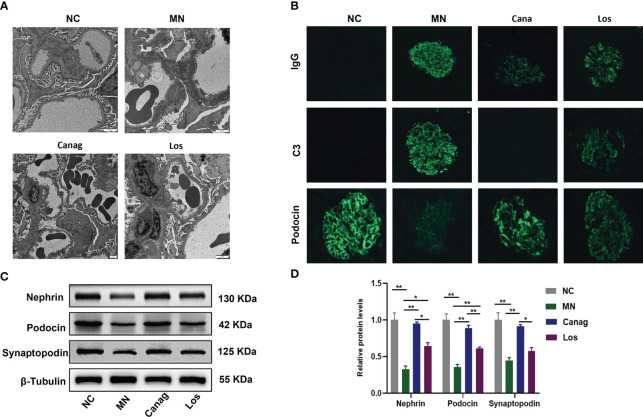
Canagliflozin inhibits renal immunity and alleviates podocyte injury in MN rats. **(A)** Representative TEM images of glomeruli. Scale bar, 2 μm. **(B)** Representative immunofluorescence images of IgG, C3, and podocin in glomeruli. Original magnification ×400. **(C, D)** Western blots and quantification analyses of nephrin, podocin, and synaptopodin. Data are shown as mean ± SEM values. One-way ANOVA followed by Tukey’s test for multiple comparisons was used. *P < 0.05, **P < 0.01.

The deposition of immune complexes is an important pathological change in IMN and an important cause of IMN. The electron microscopy results showed that the glomerular foot process injury and the deposition of electron-dense material in the subepithelial basement membrane were significantly improved in both the canagliflozin group and the losartan group after eight weeks of treatment, while the effect of canagliflozin was more obvious than losartan, as shown in **Figure 2A**. The fluorescence of glomerular IgG and C3 was significantly attenuated, and this effect was significantly stronger in the canagliflozin group than the losartan group. Our results confirm that canagliflozin significantly attenuates the deposition of immune complexes in the kidneys of MN rats. The deposition of immune complexes can directly lead to immune damage of podocytes. The expression of the podocyte markers nephrin, podocin, and synaptopodin was significantly promoted in the canagliflozin, and their expression levels were higher than those in the losartan group, all P < 0.01 (**Figures 2B–D**).

### Canagliflozin ameliorates Th1/Th2 imbalance in MN rats

**Figure 3:**
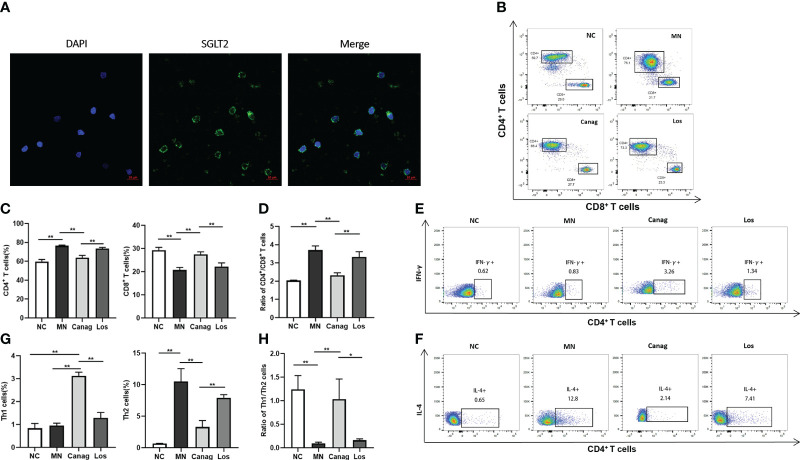
Canagliflozin regulates the imbalance in Th1/Th2 in MN rats. **(A)** Representative images showing DAPI (blue) and SGLT2 (green) staining *in vitro* T lymphocytes. **(B–D)** The number of CD4+ T-cells and CD8+ T-cells in peripheral blood PBMCs of rats detected by flow cytometry analysis. **(E–H)** The number of Th1 cells and Th2 cells in peripheral blood PBMCs of rats detected by flow cytometry analysis. Data are shown as mean ± SEM values. One-way ANOVA followed by Tukey’s test for multiple comparisons was used. *P < 0.05, **P < 0.01.

Studies have confirmed that the imbalance in Th1/Th2 cells may be an important reason why B-cells produce abnormal IgG. In order to further explore whether canagliflozin could relieve podocyte injury in MN rats through immunomodulatory mechanisms, we first examined the expression of SGLT2 in peripheral blood T-cells of rats, as shown in **Figure 3A**. Compared to the normal control group, the percentage of CD4+ T-cells in peripheral blood of MN rats increased significantly, the percentage of CD8+ T-cells decreased, and the ratio of CD4+/CD8+ increased. The change of CD4+/CD8+ ratio in MN rats was significantly reversed by eight weeks of canagliflozin treatment (3.7% ± 0.13% vs. 2.33% ± 0.08%, P < 0.01). Compared to the MN group, there was no significant difference in the CD4+/CD8+ ratio in the losartan treatment group, as shown in **Figures 3B–D**. In the MN group, the proportion of IL-4+ Th2 cells in peripheral blood increased significantly, and the ratio of Th1/Th2 had decreased significantly. Canagliflozin treatment for eight weeks not only decreased the proportion of IL-4+ Th2 cells but also increased the proportion of IFN-γ+ Th1 cells and the ratio of Th1/Th2. However, the ratio of IFN-γ+ Th1 cells and IL-4+ Th2 cells did not change significantly after losartan treatment (**Figures 3E–H**).

### Canagliflozin inhibits the secretion of IgG1 by B-cells of MN rats

**Figure 4:**
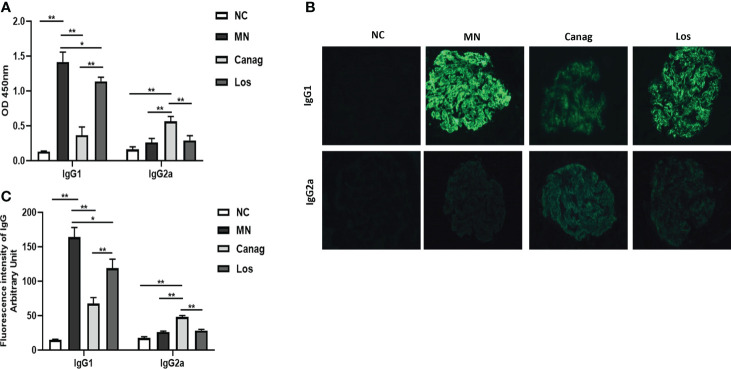
Canagliflozin decreases the secretion of IgG1 from B lymphocytes of MN rats. **(A)** Concentrations of IgG1 and IgG2a in the supernatant of B-cells were detected by ELISA. **(B)** Representative immunofluorescence images of IgG1 and IgG2a in glomeruli. **(C)** The quantitative fluorescence intensity of IgG1 and IgG2a. Data are shown as mean ± SEM values. One-way ANOVA followed by Tukey’s test for multiple comparisons was used. *P < 0.05, **P < 0.01.

On the basis of confirming that canagliflozin can significantly reverse the imbalance in Th1/Th2 cells in the peripheral blood of MN rats, we further detected the levels of IgG1 and IgG2a in supernatants of peripheral blood B-cells of rats in each group after 12 days of culture *in vitro*. The level of IgG1 produced by B-cells in the MN group increased significantly (P < 0.01), but the level of IgG2a did not change significantly. Canagliflozin significantly inhibited the secretion of IgG1 by B-cells of MN rats (0.37 ± 0.07 vs. 1.42 ± 0.08, P < 0.01) and increased the level of IgG2a in MN rats (0.56 ± 0.04 vs. 0.26 ± 0.03, P < 0.05). However, in the losartan group, the levels of IgG1 and IgG2a produced by B-cells experienced no obvious change, as shown in **Figure 4A**. Moreover, in **Figures 4B, C**, canagliflozin could significantly reduce the deposition of IgG1 in glomeruli of MN rats (67.64 ± 3.54 vs. 164.05 ± 5.75, P < 0.01) and increase the IgG2a in glomeruli (48.01 ± 0.94 vs. 26.24 ± 0.57, P < 0.01). The reduction in glomerular IgG1 in the losartan group was significantly less than that in the canagliflozin group, and there was no significant change in IgG2a compared to the MN group.

### Canagliflozin ameliorates podocyte autophagy injury induced by B-cells in MN rats

**Figure 5:**
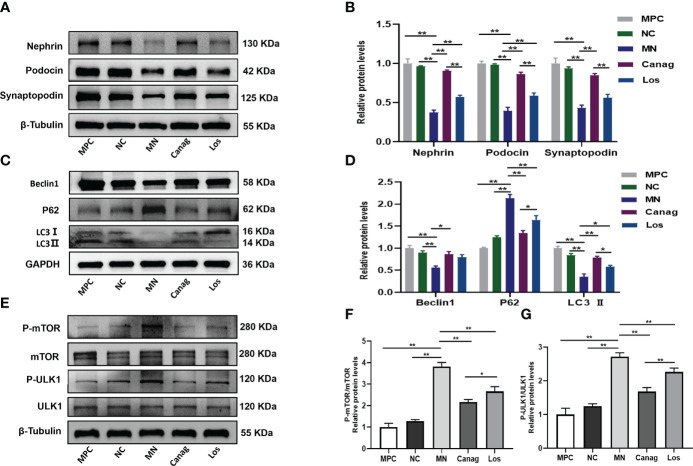
Canagliflozin ameliorates podocyte autophagy inhibiteded by B lymphocytes of MN rats. MPC group: control of normal podocytes not co-cultured with B-cells; NC group: podocytes were co-cultured with B-cells of normal control rats; MN group: podocytes were co-cultured with B-cells of MN rats; Canag group: podocytes were co-cultured with B-cells of rats treated with canagliflozin; Los group: podocytes were co-cultured with B-cells of rats treated with losartan. **(A, B)** Western blots and quantification analyses of nephrin, podocin, and synaptopodin in podocytes. **(C, D)** Western blots and quantification analyses of beclin1, p62, and LC3 II in podocytes. **(E–G)** Western blots and quantification analyses of the expression of mTOR/ULK1 in podocytes. Data are shown as mean ± SEM values. One-way ANOVA followed by Tukey’s test for multiple comparisons was used. *P < 0.05, **P < 0.01.

In order to further reveal the mechanism whereby canagliflozin reduces the deposition of immune complex and exerts reno-protective effects, we co-cultured B-cells extracted from peripheral blood of rats in each group with MPC-5 *in vitro*. Our results confirmed that co-culturing of B-cells from MN rats with podocytes inhibited the expression of nephrin, podocin, and synaptopodin in podocytes (P < 0.01) (as shown in **Figures 5A, B**). The expressions of proteins beclin1 and LC3 B, which are the autophagy markers, were also inhibited, and the expression of p62 protein was promoted (P < 0.01). Co-culturing with B-cells in the canagliflozin treatment group significantly promoted the expression of nephrin, podocin, and synaptopodin in podocytes (P < 0.01); promoted the expression of beclin1 and LC3 B proteins; and inhibited the expression of p62 protein (P < 0.05) (as shown in **Figures 5C, D**). Co-culturing with B-cells in the losartan treatment group also partially increased the expressions of nephrin, podocin, and synaptopodin in podocytes and promoted autophagy of podocytes. However, the effect of the losartan group was significantly lower than that of the canagliflozin group. The results confirmed that B-cells of MN rats could lead to impaired autophagy of podocytes, while B-cells of canagliflozin rats could improve podocyte autophagy.

The mTOR/ULK1 signaling pathway plays an important role in regulating podocyte autophagy. The mTOR(p) and ULK1(p) levels of podocytes were significantly increased after co-culture with B-cells from rats in the MN group, while the mTOR(p) and ULK1(p) levels of podocytes were significantly down-regulated by B-cells from rats in the canagliflozin treatment group (all P < 0.01), and the effect was significantly stronger than that of the losartan group, as shown in **Figures 5E–G**.

## Discussion

Idiopathic membranous nephropathy, which is a common cause of adult nephrotic syndrome, is also an important cause of renal failure. The imbalance in Th1/Th2 cells leads B-cells to produce abnormal IgG, which is an important mechanism of IMN (14, 28, 29). RAS inhibitors are currently the first-line treatment for clinical MN to lower urinary protein concentrations by reducing glomerular intracapsular pressure and improving glomerular filtration membrane function (30–32). However, RAS inhibitors have limited efficacy in patients with IMN, especially those who exhibit massive proteinuria (9, 33). SGLT2 inhibitors can also reduce glomerular intracapsular pressure through a mechanism of “tubuloglomerular feedback,” which achieves a similar effect in terms of reducing urinary protein in diabetic nephropathy (34). In addition, it has been demonstrated that SGLT2 inhibitors have reno-protective effects independent of the tubuloglomerular feedback mechanism (25). In this study, the effect of SGLT2 inhibitors on urinary protein in MN rats and the immunomodulatory mechanism independent of the tubuloglomerular feedback mechanism was first investigated.

In this study, MN rats showed mass proteinuria, hypoproteinemia, significantly increased proportions of IL-4+ Th2 cells in peripheral blood, diffuse proliferation of glomerular mesangial cells and stroma, stiff capillary loops, obvious thickening of the basement membrane, numerous spike structures, and massive IgG and C3 deposition in glomerular capillaries. These characteristics of rats confirmed that C-BSA was used to induce MN model rats successfully. In this study, we first observed the improvement of urinary protein in MN rats by canagliflozin. As expected, both of canagliflozin and losartan significantly reduced urinary protein and increased serum albumin in MN rats, but this effect of canagliflozin was better than that of losartan. This result suggests that the amelioration of urinary protein in MN rats by canagliflozin has a kidney-protection effect independent of the reduction of glomerular intracapsular pressure. The results of renal ultrasound electron microscopy showed a significant reduction in subepithelial electron-dense deposits on the podocytes of rats treated with canagliflozin, and immunofluorescence showed that canagliflozin significantly reduced the fluorescence intensity of IgG and C3 deposited along the glomerular capillary wall in granular form among the MN rats. These results confirm that canagliflozin may exert reno-protective effects by reducing the deposition of immune complexes in the kidneys.

Th1 and Th2 cells belong to 2 subpopulations of CD4+ T-cells. Thl and Th2 regulate and restrain each other and play an immunomodulatory role. Disruption of the Th1/Th2 ratio is an important cause of immune dysfunction in MN (35). Studies have shown that IMN nephropathy is an autoimmune disease closely correlated with Th2 concentrations (14, 15). The increase in Th2 cells is an important reason for abnormal IgG secretion by B-cells and immune complex deposition in the kidneys (14). Can SGLT2 inhibitors prevent B-cells from producing IgG1 by reversing the Th1/Th2 imbalance? To prove our hypothesis, we first detected the expression of SGLT receptors on T lymphocytes, and our research confirmed that SGLT2 expression on rat peripheral blood T-cells, which suggested that SGLT2 inhibitors may act directly on T cells through receptors. We further examined the changes in T lymphocyte subsets in peripheral blood PBMCs of MN rats. Flow cytometry assay showed an increase in the proportion of CD4+ T-cells in peripheral blood PBMCs of MN rats, a significant increase in IL-4–producing Th2 cells among CD4+ T-cells, and a strong polarization of both peripheral blood and renal immune responses toward Th2-type immune responses. The changes in T lymphocyte subpopulations in peripheral blood PBMCs of MN rats are identical to those in peripheral blood PBMCs of MN patients. This is consistent with previous findings (14), indicating that the imbalance of T-cell subsets plays an important role in the genesis and development of MN. There was a significant decrease in IL-4+ Th2 cells and a significant increase in IFN-γ+ Th1 cells in peripheral blood CD4+ T-cells in rats after treatment with the SGLT2 inhibitor canagliflozin. These results indicated that canagliflozin may exert a protective effect by reversing the Th1/Th2 imbalance and attenuating the deposition of subepithelial immune complexes in the glomerulus of MN rats.

Does canagliflozin exert a protective effect on podocytes by inhibiting the production of IgG secreted by B-cells mediated by homologous T-cells in MN rats? Our results show that canagliflozin significantly inhibited the level of IgG1 secretion by B-cells and attenuated IgG1 deposition in glomeruli of MN rats while promoting transformation of the IgG subtype in kidneys of MN rats to IgG2a, which indicated improvement in renal pathology (28, 29).

The deposition of immune complexes is an important mechanism leading to podocyte injury in MN. On the basis of confirming that canagliflozin can inhibit B-cell IgG1 production and renal IgG1 deposition by reversing the imbalance in Th1/Th2 cells in peripheral blood of MN rats, the mechanism by which canagliflozin can alleviate the injury of podocytes by reducing immune complexes was further explored. Studies have shown that podocyte autophagy is impaired in IMN patients (36–38). Also, the damage degree of podocyte autophagy is directly proportional to the proteinuria level of patients with IMN (39). Therefore, abnormal podocyte autophagy may be an important mechanism of podocyte injury due to immune complex deposition. We co-cultured B-cells from MN rats with podocytes and found that B-cells from MN rats significantly reduced autophagy markers beclin1 and LC3 B protein expression and significantly increased p62 protein expression in podocyte while, at the same time, decreasing the podocyte markers nephrin, podocin, and synaptopodin significantly. Our results suggested that the deposition of IgG1 is an important reason for the dysfunction of podocyte autophagy in MN rats. Co-culture of peripheral blood B-cells in rats treated with canagliflozin and podocytes significantly increased podocyte autophagy and the expression of podocyte markers and significantly reduced the damage of podocytes. Therefore, our results confirmed that canagliflozin could inhibit the secretion of abnormal IgG by B-cells and promote autophagy of podocytes in MN rats, thus reducing the injury of podocytes.

**Figure 6:**
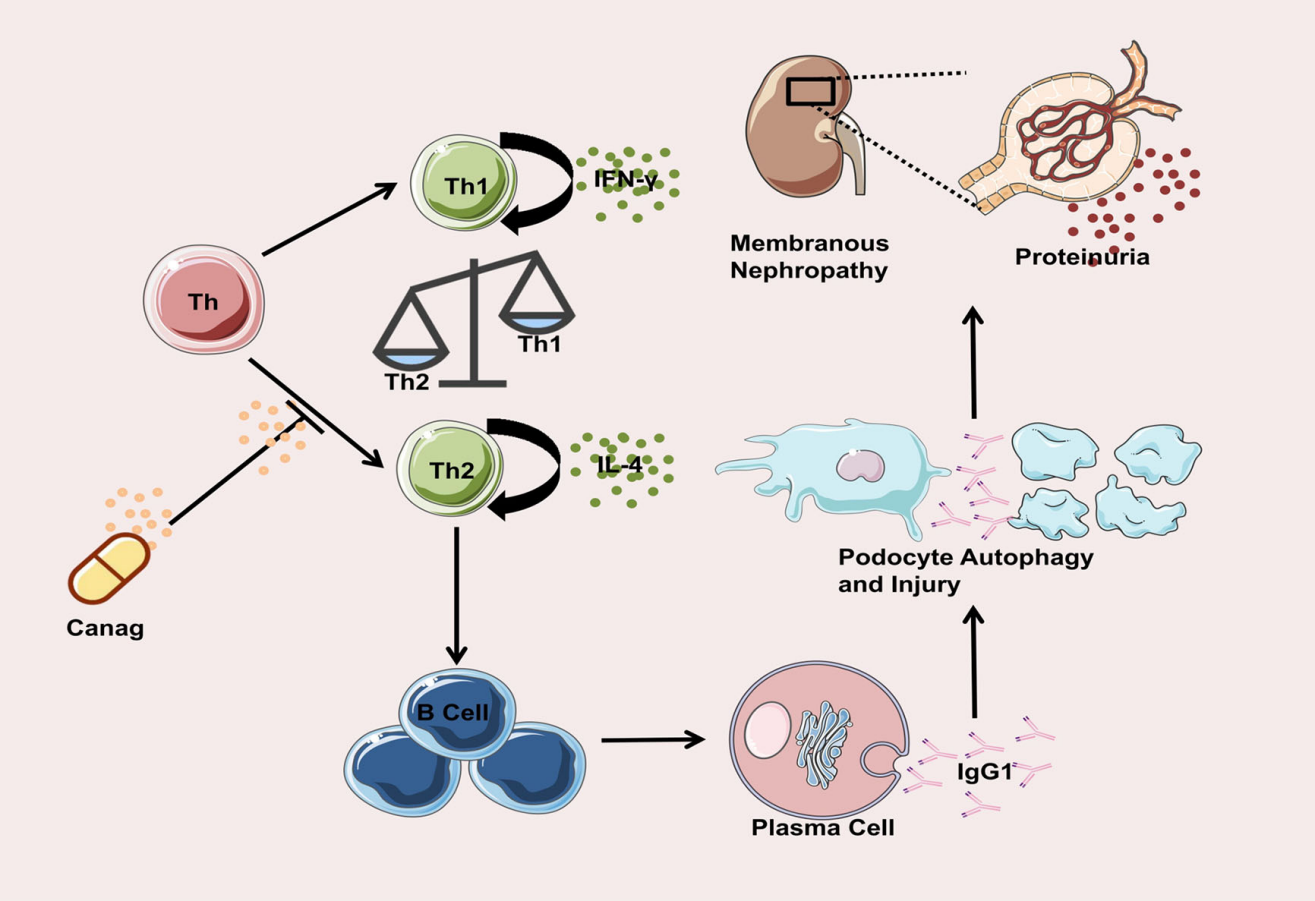
Immunomodulatory mechanism of calagliflozin in improving renal injury in MN rats.

The mTOR pathway plays an important role in the regulation of autophagy (40). Activation of the mTOR pathway down-regulates autophagy, disrupts podocyte homeostasis, and damages podocytes. Compared to the control group, the phosphorylated mTOR level of podocytes co-cultured with B-cells in the MN group significantly increased. In addition, Ser757 phosphorylation of ULK1, a member of the autophagic initial complex, was enhanced, autophagy was inhibited, and podocytes were damaged. Our results confirm that canagliflozin inhibits phosphorylation of mTOR and ULK1Ser757, suppresses activation of the mTOR/ULK1 pathway, restores the autophagy in MN rat podocytes, and improves podocyte function. Immunomodulatory mechanism of canagliflozin in improving renal injury in MN rats is shown in **Figure 6**

## Conclusion

In summary, an SGLT2 inhibitor, canagliflozin, exerted a protective effect on kidneys by reversing the Th1/Th2 cell imbalance and attenuating the impairment of autophagy of podocytes mediated by abnormal IgG1 secretion in MN rats. C-BSA induced MN model cannot fully mimic human IMN, whether SGLT2 inhibitors have the renoprotective effect on IMN and its underlying mechanism still needs further study.

## Data availability statement

The raw data supporting the conclusions of this article will be made available by the authors, without undue reservation.

## Ethics statement

The animal experiments were approved by the Ethics Committee of Chu Hsien-I Memorial Hospital of Tianjin Medical University (Approval No. DXBYY-IACUC-2021044).

## Author contributions

PY and SZ conceived of the design of the study and edited of the manuscript. XL and JW performed the experiments, analyzed the data, and drafted the manuscripts. LZ, XS, YL, HL, GM and JL provided acquisition, analysis, interpretation of data and statistical analysis. All of the authors have read and approved the final manuscript. All authors contributed to the article and approved the submitted version.

## Funding

This study was supported by the following grants and funding sources: National Natural Science Foundation of China (no. 81600643), Tianjin Science and Technology Support Project (no. 18ZXZNSY00280, 21ZXGWSY00100), Science and Technology Foundation of Tianjin Health and Health Commission (no. ZC20128), Key social science projects of the Tianjin Education Commission (no. 2019JWZD54), Scientific Research Funding of Tianjin Medical University Chu Hsien-I Memorial Hospital (no. 2018ZDKF05), and Fundamental Research Program of Shanxi Procince (No. 20210302124569).

## Acknowledgments

We would like to thank all the members in Chu Hsien-I Memorial Hospital & Tianjin Institute of Endocrinology for all their assistance during this project. We also thank Core Facility of Research Center of Basic Medical Sciences (Tianjin Medical University) for flow cytometry analyses.

## Conflict of interest

The authors declare that the research was conducted in the absence of any commercial or financial relationships that could be construed as a potential conflict of interest.

## Publisher’s note

All claims expressed in this article are solely those of the authors and do not necessarily represent those of their affiliated organizations, or those of the publisher, the editors and the reviewers. Any product that may be evaluated in this article, or claim that may be made by its manufacturer, is not guaranteed or endorsed by the publisher.
